# P80 Natural Essence Exerts Efficient Anti-HIV-1- as Well as Adjuvant Effects in DCs

**DOI:** 10.3390/vaccines9090976

**Published:** 2021-08-31

**Authors:** Viktoria Zaderer, Wilfried Posch, Ronald Gstir, Przemyslaw A. Filipek, Günther K. Bonn, Pornanong Aramwit, Lukas A. Huber, Doris Wilflingseder

**Affiliations:** 1 Institute of Hygiene and Medical Microbiology, Medical University of Innsbruck, 6020 Innsbruck, Austria; viktoria.zaderer@i-med.ac.at (V.Z.); wilfried.posch@i-med.ac.at (W.P.); 2 ADSI—Austrian Drug Screening Institute GmbH, 6020 Innsbruck, Austria; ronald.gstir@adsi.ac.at (R.G.); przemyslaw.filipek@adsi.ac.at (P.A.F.); Guenther.Bonn@adsi.ac.at (G.K.B.); lukas.a.huber@i-med.ac.at (L.A.H.); 3 Department of Pharmacy Practice, Faculty of Pharmaceutical Sciences and Center of Excellence in Bioactive Resources for Innovative Clinical Applications, Chulalongkorn University, Bangkok 10330, Thailand; aramwit@gmail.com; 4 Institute of Cell Biology, Biocenter Innsbruck, Medical University of Innsbruck, 6020 Innsbruck, Austria

**Keywords:** HIV-1, complement, dendritic cells, P80 natural essence

## Abstract

Dendritic cells (DCs), as well as complement, play a major role during human immunodeficiency virus 1 (HIV-1) entry and infection at mucosal sites. Together, DCs and complement are key points for understanding host defence against HIV-1 infection and for studying the impact of new drugs on the regulation of innate host-pathogen interactions and adaptive immunity. For this, we evaluated the antiviral effect of the P80 natural essence (Longan extract) on interactions of non- and complement-opsonized HIV-1 with DCs. In viability assays, we first illustrated the effects of P80 natural essence on DC function. We found that P80 concentrations above 1.5% caused increased cell death, while at concentrations between 0.5% and 1% the compound exerted efficient antiviral effects in DCs and illustrated an adjuvant effect regarding DC activation. DC maturation, as well as co-stimulatory capacity, were significantly improved by P80 natural essence via p38 MAPK phosphorylation in presence of the viral challenge independent of the opsonization pattern. These findings might be exploited for future therapeutic options to target DC subsets directly at mucosal sites by P80 natural essence and to block entry of both, non- and complement-opsonized HIV-1.

## 1. Introduction

Although chronic viral diseases, such as HIV-1 or HCV, can be treated very efficiently, the use of drugs such as RT or protease inhibitors is associated with significant side effects and very high treatment costs. Thus, there is a need to develop safe, effective and inexpensive drugs for the treatment of these infections. The subtropical and tropical Longan plant (*Dimocarpus longan* Lour.) belongs to the Sapindaceae family and is economically important in terms of being used in the fresh fruit industry, as dried longan pulp, longan juice, longan jelly, longan wine, canned longan in syrup as well as in medical approaches as a potential immunomodulatory agent [1,2,3,4,5,6].

Incoming pathogens are recognized by antigen-presenting cells such as dermal DCs, Langerhans Cells (LCs) or macrophages via an array of pattern-recognition receptors (PRRs), including Toll-like receptors (TLRs), C-type lectin receptors (CLRs) and importantly complement receptors (CRs). CRs are abundantly expressed on DCs, and the significance of complement with respect to inducing innate and adaptive immunity in response to pathogens via intrinsic or receptor-activating functions was highlighted recently [7,8,9,10]. When exposed to HIV-1 surrounded by covalently linked complement C3 fragments DCs were shown to produce innate cytokines including type I interferons (IFN-α, IFN-β), interferon-stimulated genes (ISGs), pro-inflammatory cytokines (IL-1β, IL-6, IL-23) and the complement anaphylatoxin C3a [8,9]. Such complement-opsonized HIV-1 (HIV-C) in addition mediated significantly enhanced DC infection, DC maturation and stimulation of efficient HIV-1-specific CTL responses [8,11]. In contrast, non-opsonized HIV-1 (HIV) was illustrated to manipulate DCs to mediate only partial DC maturation and to efficiently transmit the virus to target T cells via the virological synapse [12]. This partial DC maturation by HIV resulted in migration and enhanced DC/T cell interactions, but lacked efficient antiviral activity [13]. Here, we have investigated the antiviral activity and DC boosting capacity of a crude extract from Longan extract (P80 natural essence) during exposure to non- or complement-opsonized HIV-1. Notably, the P80 natural essence exerted an adjuvant role during exposure of DCs to both, non- and complement-opsonized HIV-1, and significantly increased DC maturation also for non-opsonized HIV-1. Further, it completely abrogated infection of DCs with both, HIV and HIV-C, despite neither mediating virolysis nor lowering viral binding to the most prominent antigen-presenting cells. The antiviral and DC-boosting effects were further accompanied by P80-mediated MAPK modulations in DCs. Therefore, it can be assumed from our in vitro analyses that the Longan natural essence could be applied as a mucosal adjuvant to provide a link between innate and adaptive immunity by exerting immune-stimulating effects on antigen-presenting cells. Moreover, natural products from medicinal plants represent an attractive alternative approach to exerting anti-HIV-1 effects and additionally, our findings suggest, that P80 natural essence may be useful for boosting the immune-stimulatory capacity of DCs during HIV-1 infection.

## 2. Materials and Methods

### 2.1. Longan Extract

Longan extract (P80 natural essence) is a herbal extract from PM 80 Co., Ltd., Bangkok, Thailand (herbal registration number i6200134/62).

### 2.2. Ethics Statement

The use of anonymized specimens for scientific purposes was approved by the Ethics Committee of the Medical University of Innsbruck, and informed consent was obtained from all volunteer blood donors by the Central Institute for Blood Transfusion & Immunological Department, Innsbruck, Austria (ECS1166/2018, 14 November 2018).

### 2.3. Generation of Human Monocyte-Derived DCs

Blood for the monocyte isolation was received by the Central Institute for Blood Transfusion and Immunological Department, Innsbruck, Austria. Briefly, PBMCs (peripheral blood mononuclear cells) were isolated from the blood of healthy donors [8,14,15,16] obtained by a density gradient centrifugation using a Ficoll Paque Premium (GE Healthcare) gradient. After washing, CD14+ monocytes were isolated from PBMCs using anti-human CD14 Magnetic Beads (BD)—The purity of the isolated cells was at least 98%. Monocytes were stimulated by the addition of IL-4 (200 U/mL) and GM-CSF (300 U/mL) for 5 days to generate iDCs, which were used for all further experiments. Non-stimulated iDCs were used as controls for all experiments using DCs.

### 2.4. Multicolor FACS Analyses

Differentially treated DCs were analysed by using anti-human CD11b-PE, CD11c-AlexaFluor488, CD18-APC, HLA-DR-APC-Cy7, DC-SIGN-PE, CD86-FITC, CD83-APC, as described [14] on a FACS Verse flow cytometer (BD Biosciences, Franklin Lakes, NJ, USA). Data were analysed using FACS DIVA software (BD Biosciences, Franklin Lakes, NJ, USA).

### 2.5. DC Infection

Cells were infected in triplicates using differentially opsonized HIV-1 plus/minus treatment using varying concentrations of P80 natural essence as described before [8,15]. Briefly, cells (1 × 10^5^/100 μL) were treated or not with P80 natural essence 1 h pre- or post-infection and incubated for 3 h with HIV or HIV-C (50 ng p24/mL) or left uninfected and virus concentrations from supernatants were measured on 7 dpI. To confirm productive infection by HIV-1 and not a cell-associated virus, we thoroughly washed the cells after overnight incubation with different viruses and cultured the cells at 37 °C/5% CO_2_. By ELISA we measured the p24 concentrations of the supernatants following spinning down the plate to pellet cells on 7 dpI.

### 2.6. Immunoblot Analyses of Phosphorylated Proteins

DCs were starved in RPMI 1640 containing 0.5% FCS and 1% L-Glutamine for 3 h. Starving of cells was performed to set their phosphorylation to background levels. Following starvation, DCs were incubated with P80 natural essence and differentially opsonized HIV-1 particles. After 4 h co-incubation, cells were lysed with RIPA Buffer (Sigma-Aldrich, St. Louis, MI, USA) containing protease and phosphates inhibitors and EDTA (Thermo Fisher Scientific, Waltham, MA, USA) for 20 min at 4 °C. The protein content was determined by BCA (Thermo Fisher Scientific, Waltham, MA, USA). Lysates were separated using 10% SDS-PAGE gels, transferred to PVDF membranes and incubated with anti-human p38 MAPK as loading control as well as anti-human phospho-p38 MAPK (1:1000, Cell Signaling Technology, Danvers, MA, USA) and developed with the Lass 4000 Image Quant. For this, the peak values of the target protein were divided by the peak values of the loading control before doing a relative comparison. Quantification was performed using values from three to six different experiments.

### 2.7. p24 ELISA

To analyse supernatants for HIV p24 capsid protein, a p24 sandwich ELISA was performed as described [8]. Briefly, the ELISA plate was coated using a mouse MAb against HIV-1 p24 Ag (200 ng/well). For infection experiments lysed samples (diluted 1:1 with 2% Igepal) were added for 1 h at room temperature, the virolysis assay is described below. Bound HIV-1 p24 Ag was detected with a second biotinylated anti-p24 MAb followed by streptavidin–β-galactosidase conjugate. The colour reaction was developed with the resorufin-β-d-galactopyranoside substrate (Sigma, St. Louis, MO, USA), and the optical density was measured on an ELISA microplate reader at 550 nm. p24 amounts were calculated from a standard curve by using recombinant HIV-1 p24 Ag. All reagents for p24 ELISA were kindly provided by Polymun Scientific, Austria.

### 2.8. Virolysis

To analyse, if P80 directly causes lysis of HIV-1, we performed a virolysis assay described by Bánki et al., 2005 [16]. Briefly, HIV or HIV-C (250 ng p24/mL) were incubated in either detergent (2% Igepal) as a positive control for virolysis or at various P80 concentrations (0.1% to 10%) in a 96-well plate in triplicates for 24 h at 37 °C/5% CO_2_. The solutions were added to a pre-coated p24 ELISA plate the next day and incubated for 1 h at RT on a shaker (300 rpm). The non-bound, non-lysed virus was removed by washing the plates five times with medium, the bound virus was lysed in 2% Igepal and quantified by p24 ELISA.

### 2.9. Statistical Analysis

Differences were analysed by using GraphPad Prism software (GraphPad Software Inc., California, CA, USA) and one-way ANOVA with Bonferroni post-test for multiple comparisons or Unpaired Student’s *t*-test depending on the analyses performed.

## 3. Results

### 3.1. P80 Natural Essence Concentrations above 1% Result in Decreased DC Viability

**Figure 1:**
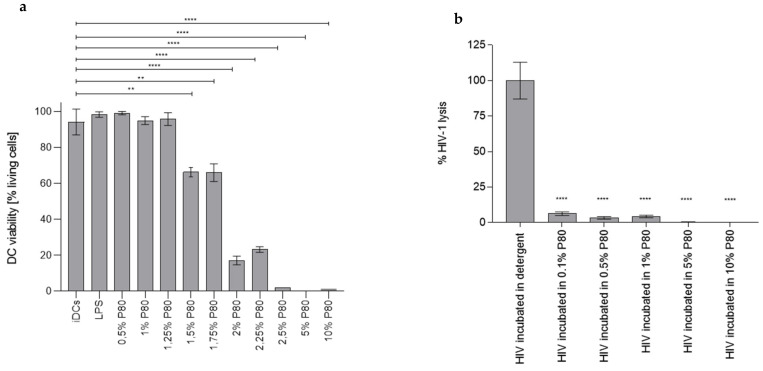
(**a**) DC viability is not affected up to 1.25% P80 natural essence. DCs exposed to various P80 natural essence concentrations (0.25–10%) or LPS as a positive control for DC stimulation were analysed for their viability on 2 days post-infection (dpI). P80 concentrations 1.5% to 10% significantly decreased DC viability. These data were obtained in three independent experiments and a summary of all experiments is illustrated. Statistical differences were analysed using GraphPad Prism software and one-way ANOVA with Dunnett’s post-test. ** *p* < 0.01, **** *p* < 0.0001 (**b**) P80 natural essence does not induce viral lysis. HIV-1 (1 µg p24/mL) exposed to various P80 natural essence concentrations (0.1–10%) was analysed for viral lysis induced by P80. As illustrated below, P80 had only little effect on the virus and the virolysis induced by all P80 concentrations was significantly lower (*p* < 0.0001) compared to the detergent control set as 100%. The experiment was repeated three times independently and a summary is depicted. Statistical differences were analysed using GraphPad Prism software and one-way ANOVA with Dunnett’s post-test. **** *p* < 0.0001.

First, we monitored, whether the viability of human immune cells such as dendritic cells (DCs) was affected by P80 natural essence. For this, monocyte-derived DCs or PBMCs were exposed to various P80 natural essence concentrations (0.25–0.5–0.75–1–1.25–1.5–1.75–2–2.25–2.5–5–10%) for 2 days. Following fixation, cells were stained using Live/Dead staining and analysed using multi-colour flow cytometry. As illustrated in Figure 1a, the P80 natural essence only had minor effects on DC viability at a concentration range from 0.25% to 1.25%. From 1.5% and above, the P80 natural essence exhibited significant cytotoxic effects on DCs after 2 days’ incubation (Figure 1a). The DC viability of P80-treated samples was comparable to iDCs and LPS-exposed DCs, which are routinely used as positive controls for DC stimulation (Figure 1a). These experiments revealed that P80 natural essence concentrations above 2.5% affected DC viability.

### 3.2. P80 Natural Essence Does Not Induce Virolysis

Next, we investigated, whether effects exerted on DC by P80 natural essence during infection with HIV-1 were due to lysis of the virus. HIV-1 (1 µg p24/mL) samples were incubated for 1 h in either detergent (2% Igepal [=NP-40]) as positive control for complete viral lysis (set as 100% virus lysis) or increasing concentrations of P80 (0.1–10%). After the incubation time of the virus in detergent or various P80 concentrations, samples were transferred to a pre-coated p24 ELISA plate and incubated for 1 h shaking to allow binding of free p24 Ag. Thereafter, the ELISA plate was thoroughly washed to remove unbound Ag. Bound p24 Ag—corresponding to the lysed virus—was determined by standard p24 ELISA detection as described in the Methods section. As illustrated in Figure 1b, P80 had only little effect on the virus and the virolysis induced by all P80 concentrations was significantly lower (*p* < 0.0001) compared to the positive control. Data showed that P80 natural essence did not have any virolytic effects on HIV-1.

### 3.3. Binding of HIV Is Not as Affected by P80 Natural Essence as HIV-C attachment

**Figure 2:**
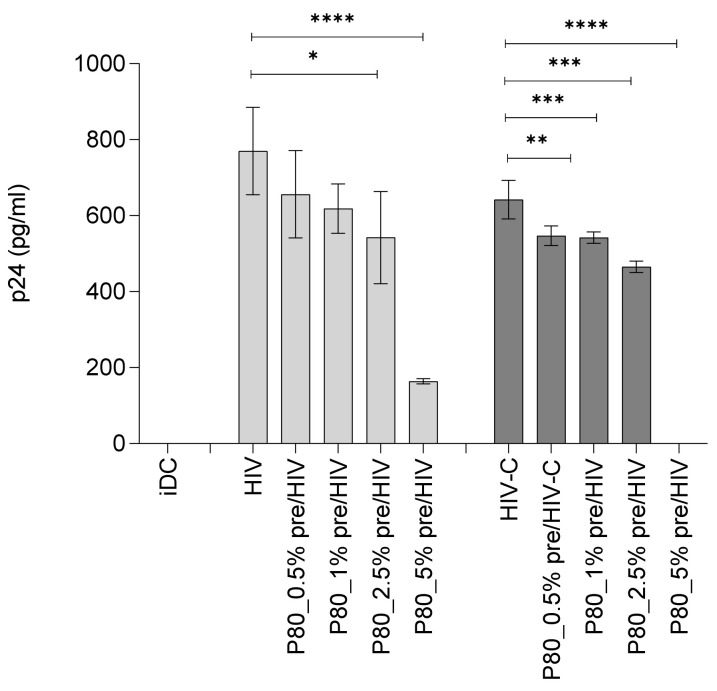
The binding of HIV and HIV-C to DCs is differentially affected by the P80 natural essence. HIV-1 binding to DCs was not significantly changed by pre-incubation of cells using 0.5% and 1% P80 natural essence, while HIV-C binding was significantly decreased in these concentrations. At P80 concentrations above 2.5%, a highly significant reduction in HIV and HIV-C binding to DCs was detected (HIV, light grey bars; HIV-C, dark grey bars). Uninfected iDCs were used as negative controls. Data were obtained in three independent experiments and a summary of all experiments is illustrated. Statistical analyses were done using GraphPad Prism software and one-way ANOVA with Dunnett’s post-test. * *p* < 0.05, ** *p* <0.01, *** *p* < 0.001, **** *p* < 0.0001.

To monitor, if binding of non-opsonized (HIV) or complement-opsonized HIV-1 (HIV-C) to DCs was affected in the presence of P80, concentrations of P80 not affecting DC viability (0.5 and 1%) as well as affecting its viability (2.5% and 5%) were applied to the cells 30 min prior addition of HIV-1 (Figure 2). These P80 concentrations were chosen to assign possible effects on DC viral loads to either toxicity (>1.25%) or interference with binding capacities (<=1.25%) in the next step (see below). Binding was performed for 3 h at 4 °C to allow virus attachment, but not internalization. We found that binding of HIV was not significantly lower in presence of low P80 concentrations (0.5% and 1%), while 2.5% and 5% caused a significant reduction in viral binding (Figure 2, light grey bars). In contrast, HIV-C binding to DCs was significantly hampered at all concentrations tested (Figure 2, dark grey bars) indicating an impeded binding of complement-opsonized HIV to DCs. These analyses revealed that P80 natural essence decreases binding of HIV and HIV-C to DCs, with HIV-C being more affected compared to HIV.

### 3.4. Productive Infection of DCs by Both, HIV and HIV-C, Is Abrogated by Pre-Incubation with 1% P80 Natural Essence

**Figure 3:**
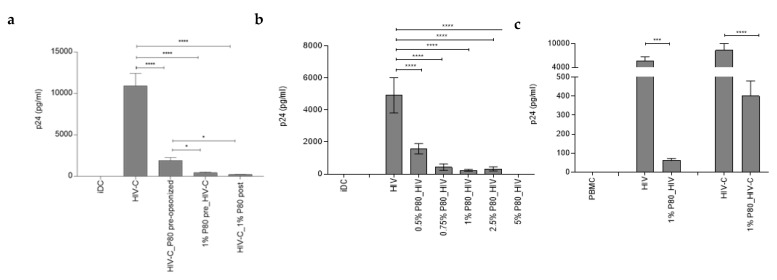
(**a**) Productive HIV infection is abrogated by pre-incubation with 0. 5% to 5% P80 natural essence. DCs (1 × 10^5^/100 µL) were pre-incubated with 0.25 to 1% P80 natural essence for 1 h prior infection with non-opsonized HIV-1 (50 ng p24/mL). After 7 days, cell-free supernatants were collected and a p24 ELISA was performed in triplicates. The essence highly significantly blocked HIV infection at all concentrations tested. Uninfected iDCs were used as negative controls. Data were obtained in three independent experiments in triplicates and a representative experiment is depicted. (**b**) P80 natural essence blocks DC infection of complement-opsonized HIV-1 independent of the mode of addition. DCs were loaded with either HIV-C that was opsonized with 1% P80 prior addition (HIV-C_P80 pre-opsonized, cells were pre-incubated with 1% P80 prior HIV-C addition (1% P80 pre_HIV-C) or infected with HIV-C prior addition of 1% P80 (HIV-C_1% P80 post). Compared to high DC infection using HIV-C, all treatments highly significantly reduced productive infection in DCs. (**c**) Productive PBMC infection is significantly impaired upon pre-incubation with 1% P80 natural essence. IL-2-stimulated PBMCs (1 × 10^5^/100 µL) were pre-incubated with 1% P80 natural essence for 1 h prior infection with differentially opsonized HIV-1 (50 ng p24/mL). After 5 days, cell-free supernatants were collected and a p24 ELISA was performed in triplicates. Uninfected PBMCs were used as negative controls. Data were obtained in three independent experiments in triplicates and a summary is shown. For all experiments, statistical differences were analysed using GraphPad Prism software and one-way ANOVA with Dunnett’s post-test. * *p* < 0.05, *** *p* < 0.001 **** *p* < 0.0001.

Further, productive DC infection was analysed in the absence and presence of varying P80 concentrations (0.5% to 5%) in three independent donors. First, cells were pre-incubated or not with P80 prior to infection with non-opsonized HIV-1 (Figure 3a, HIV). At all concentrations tested, the compound exerted a strong antiviral effect with—Only at 0.5%—Signs of productive DC infection on 7 dpI (Figure 3a). Since complement-opsonized HIV-1 illustrated a significantly higher DC infection compared to non-opsonized HIV [8,9,14], we next applied 1% of P80 natural essence to DCs 1 h prior (Figure 3b, 1% P80 pre_HIV-C) or 1 h after infection with HIV-C (Figure 3b, 1% P80 post_HIV-C). In addition, we treated the complement-opsonized virus preparation with 1% P80 prior to loading DCs (Figure 3b, HIV-C_P80 pre-opsonized). DCs were then cultured for 7 days at 37 °C/5% CO_2_. Cell-free supernatants were collected at 7 dpI and p24 ELISA was performed. Compared to the already described efficient productive infection of DCs with HIV-C, all P80 treatments (Figure 3b) significantly blocked productive DC infection, with pre-and post-treatment being the most effective. Similar results were obtained using non-opsonized HIV-1 (Figure S1). To verify the effects of the P80 natural essence also in directly isolated PBMCs, we pre-incubated IL-2-stimulated PBMCs with 1% P80 prior to infection with HIV or HIV-C. p24 values were measured on 5 dpI. Both, productive infections with HIV and HIV-C were significantly impaired in primary PBMCs (Figure 3c). p24 values dropped from 5580 +/− 1100 to 60 +/− 12 and 8350 +/− 1650 to 400 +/− 80 for HIV or HIV-C, respectively, in three independent donors. These analyses revealed that the P80 natural essence exerted a potent antiviral effect regarding DC and PBMC infection independent of the opsonization pattern of the virus.

### 3.5. P80 Natural Essence Augments Co-Stimulatory Capacity and Maturation in HIV- and HIV-C-Exposed DCs

**Figure 4:**
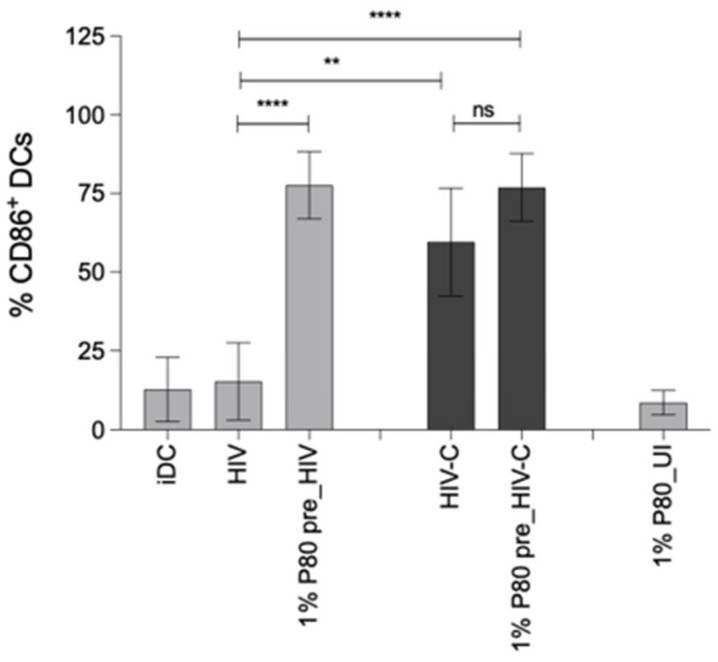

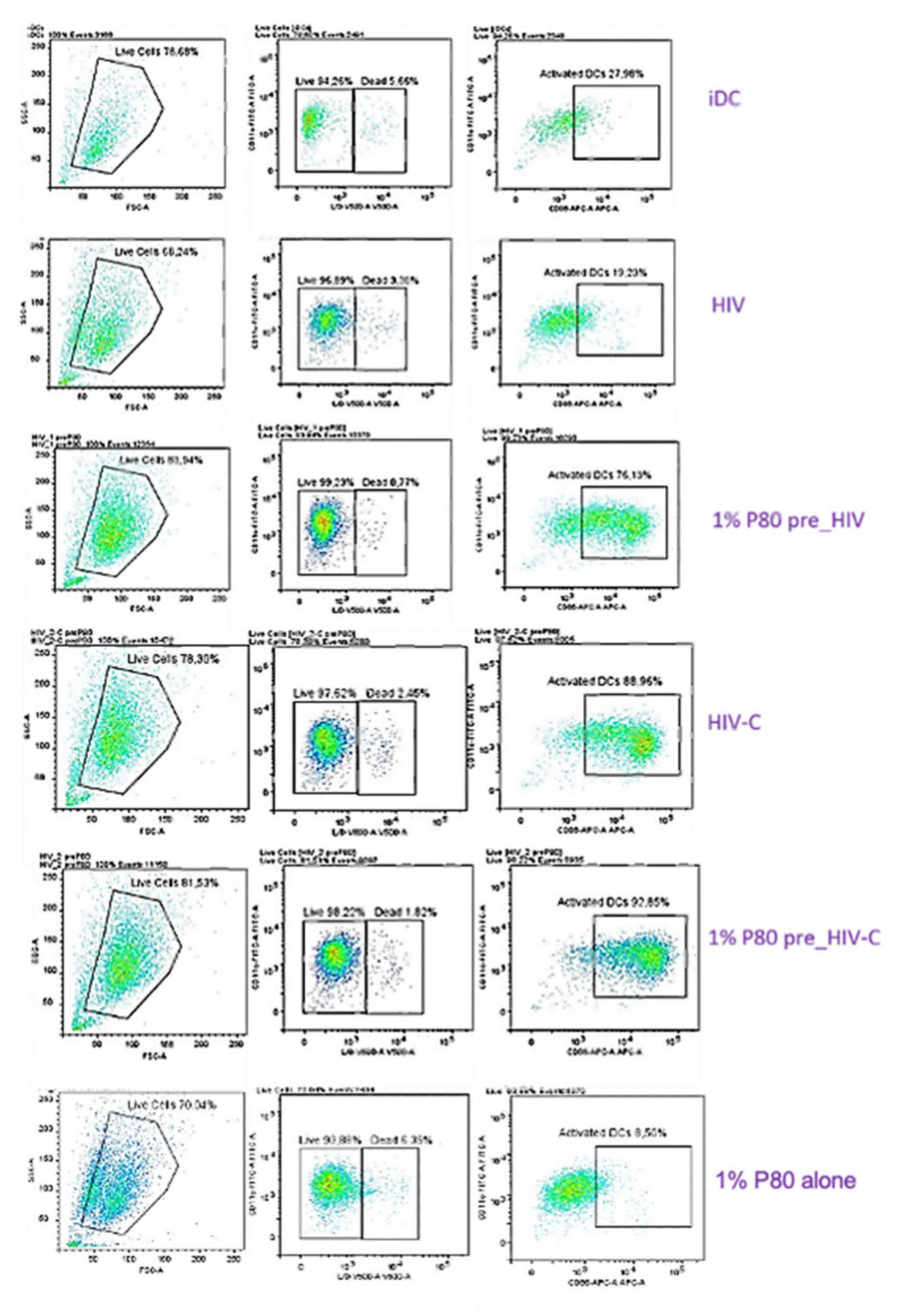
P80 natural essence boosts maturation and co-stimulatory capacity of HIV- and HIV-C-exposed DCs. HIV-loaded DCs illustrate significantly lower CD86 expression compared to HIV-C-exposed DCs (HIV vs. HIV-C). Pre-incubation of DCs with 1% P80 natural essence boosts the co-stimulatory capacity of HIV- (left) and HIV-C-(right) loaded DCs (1% P80 pre_HIV, 1% P80 pre_HIV-C), while in P80-incubated DCs this effect was not observable (1% P80 alone). The upper panel shows a summary of 4 independent donors, the lower one representative donor and the gating strategy. Dead cells were excluded by live/dead staining and activated DCs were gated as CD11chigh/CD86high population. Statistical significance was calculated with GraphPad Prism software and one-way ANOVA with Dunnett’s post-test. ** *p* < 0.01, **** *p* < 0.0001. UI, uninfected.

Efficient DC infection using HIV-C was associated with enhanced DC maturation and co-stimulatory capacity [8]. Since the P80 natural essence antagonized productive infection of DCs, we investigated the maturation and co-stimulatory capacity of HIV- and HIV-C-exposed DCs in presence of this natural compound. For this, DCs were loaded with non- or complement-opsonized HIV in the absence and presence of 1% P80 natural essence (Figure 4). As described earlier [8], we also found that HIV-C mediated a significantly higher DC maturation as assessed by upregulation of the co-stimulatory marker CD86 compared to HIV (Figure). Surprisingly, despite completely suppressing DC infection, 1% P80 natural essence significantly enhanced CD86 surface expression in HIV-exposed DCs and even slightly increased this marker also in already CD86-high HIV-C-loaded DCs. In Figure 4, upper panel, a summary of 4 independent experiments is shown, the lower panel depicts one representative donor exposed to HIV or HIV-C in the absence and presence of 1% P80. Immature DCs (iDC) were used as negative controls (Figure 4). In addition to CD86, also the maturation marker CD83 was expressed to higher levels in P80-treated conditions, independent of opsonization of virus (Figure S2, non-opsonized HIV plus (grey)/minus (green) 1% P80 pre-stimulation. iDC (blue) or P80 only-treated DCs (yellow) served as negative controls. These analyses revealed that P80 served as a natural adjuvant for DC maturation and co-stimulatory capacity.

### 3.6. P80 Natural Essence Modifies MAPK Signaling in HIV- and HIV-C-DCs

**Figure 5:**
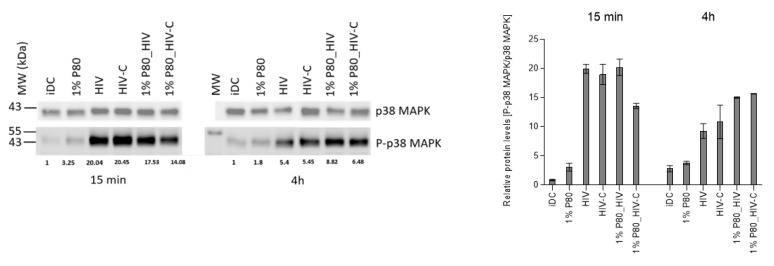
P80 natural essence activates sustained p38 MAPK phosphorylation in HIV- and HIV-C-exposed DCs. (**Left**) HIV- and HIV-C-loaded DCs illustrate pre-treated with 1% P80 natural essence illustrate increased p38 MAPK phosphorylation compared to their infected counterparts (HIV vs. HIV-C) after 4 h. iDCs and DCs treated with 1% P80 only were used as negative controls, p38 MAPK phosphorylation (P-p38 MAPK) was normalized to the loading control (p38 MAPK) and iDCs at the respective time point (15 min, left; 4 h right). x-fold increase in the P-p38 MAPK signal is illustrated below the IB. (**Right**) A summary of p38 MAPK phosphorylation at 15 min and 4 h of three independent experiments is illustrated.

p38 MAPK phosphorylation was shown previously to be associated with DC maturation [17]. To gain insight into the signalling pathway involved in DC activation following P80/HIV or HIV-C treatment and, we next examined p38 MAPK activation in short- (Figure 5, 15 min) and sustained (Figure 5, 4 h) experimental settings. Immunoblot analyses revealed, that despite abrogated productive DC infection, pre-treatment with 1% P80 (1% P80_HIV, 1% P80_HIV-C) mediated more efficiently p38 MAPK phosphorylation in long-term experiments (4 h) compared to their infected counterparts (HIV, HIV-C), while this phosphorylation was more pronounced in short-term experiments in HIV- and HIV-C-exposed DCs compared to their P80-treated counterparts. In Figure 5, a representative immunoblot and on the right a summary of three independent experiments is illustrated. These data revealed a sustained p38 MAPK phosphorylation in P80-treated in HIV- and HIV-C-loaded DCs.

## 4. Discussion

The benefits of using naturally-derived medicinal plants for the treatment of infectious diseases are multifaceted, from their high abundance, low or no side effects to their bioavailability. Out of ~27 million plant species on our planet, only a few compounds have been tested so far for their antiviral properties against HIV-1 (rev. in [18]). Here, we investigated antiviral and DC-modulating capacities of the subtropical and tropical Longan plant (*Dimocarpus longan* Lour.). We illustrated an adjuvant effect on DC functions by P80 natural essence by upregulating DC maturation and co-stimulatory markers. In addition, the P80 natural essence interfered with productive DC and PBMC infection by non- or complement-opsonized HIV-1. The reduced DC infection after in particular exposure to HIV-C that was detected using 0.75% and 1% P80 natural essence resulted from decreased binding of the virus preparation, while at higher concentrations it was due to toxicity caused by the P80 natural essence.

Recently, we found that complement-opsonization of HIV-1 was able to relieve restriction mechanisms in DC, thereby initiating strong DC maturation, the co-stimulatory capacity of DCs and stimulation of efficient cellular and humoral antiviral immunity [8]. In contrast, non-opsonized HIV-1 evolved mechanisms to avoid efficient antigen-presentation via DCs, the sentinels of our immune system [19,20], and if these sensors were bypassed as observed with complement-opsonized HIV-1, an efficient antiviral immune response was induced. DCs are relatively resistant to productive infection with HIV-1 due to several restriction mechanisms [20,21,22] and low DC infection with HIV-1 resulted in missing DC maturation, co-stimulation and type I interferon responses. In contrast, when Manel et al. co-delivered HIV-2 or SIV Vpx together with HIV-1 into DCs, a significantly enhanced DC infection, activation and induction of antiviral immunity were observed [20]. We found that the natural essence P80 from the Longan plant completely abrogated productive DC infection at concentrations as low as 0.75%, even if exposing the cells to highly infectious HIV-C preparations overcoming restriction in DCs [8]. Low-level productive DC infection with HIV-1 was also impaired when cells were pre-incubated with the natural essence. Similar results were obtained with IL-2-stimulated PBMCs, and treatment with P80 natural essence also inhibited productive infection in such cultures.

Despite omitting productive DC infection with both, non- and complement-opsonized HIV-1, the P80 natural essence exerted a strong adjuvant effect on DC maturation and up-regulation of the co-stimulatory molecule CD86. These features were significantly increased in P80-treated and HIV-exposed DCs and this effect was even higher in HIV-C-exposed DCs. Thus, the P80 natural essence seemed to trigger an activation program in HIV-1-exposed DCs independent of the opsonization pattern of the virus. This was further confirmed by sustained p38 MAPK phosphorylation in P80-treated and HIV- or HIV-C-infected DCs, pointing to a possible role of P80-mediated p38 MAPK activation for enhancement of DC maturation. p38 MAPK and NFκB activation have been shown to being pivotal in the DC maturation process as blocking one of these signalling pathways inhibited LPS-induced maturation of DCs [23,24]. Activation of NFκB, p38 MAPK and ERK1/2 was also essential in the significantly improved DC functions and adjuvant effects observed by complement opsonization of HIV-1 [8,9,11]. The association of P80-mediated sustained MAPK phosphorylation with DC maturation and activation as well as the CTL-stimulatory capacity of P80-/HIV-exposed DCs has to be characterized in detail in a follow-up study. The P80-mediated antiviral effect observed in DCs in terms of complement-opsonized HIV-1 is critical as very recently Nijmeijer et al. [25] illustrated that complement present in semen enhances HIV-1 infection of Langerhans Cells (LCs) that are the first immune cells encountering the virus during sexual contact. This process was mediated via binding to complement receptors 3 and 4 (CR3, CR4) and bypassing of langerin, thereby changing the restrictive nature of LCs into virus-disseminating cells [25] Targeting LCs and dermal DCs by P80 natural essence, which shows an antiviral as well as the antigen-presenting cell-boosting mechanism, provides thus a new tool to effectively prevent HIV-1 transmission at mucosal surfaces. Moreover, plant-based HIV-VLPs were described that used plants for recombinant expression of HIV antigens to target mucosal immune responses [26]. *Nicotiana (N.) benthamiana* was used to stably express HIV gag p55 and deconstructed (d) gp41 (dgp41), comprising the HIV MPER, transmembrane domain, and cytoplasmic tail in transgenic gag plants [27]. Mice primed/boosted with plant VLP showed high levels of anti-gag serum IgG and lower levels of anti-gp41 responses. VLPs were supplemented with Ribi adjuvant and these data suggested that an adjuvant will be needed for a good mucosal targeting strategy. Here, the P80 natural essence is promising to optimally stimulate antigen-presenting cells at mucosal sites. Another possible application of the P80 natural essence is as topical microbicide due to its strong antiviral properties exerted in vitro. Of course, the in vitro data are just a first step in a number of future testing—obviously, mucosal distribution, concentration, achieving a sufficiently high concentration of P80 in target cells and retention are critical parameters. As applying the P80 natural essence directly at mucosal surfaces to target DCs and LCs, the concentrations used in our in vitro setting with a range of 0.5 to 1% might be achieved in an in vivo scenario, but this needs further confirmation in an in vitro 3D mucosal tissue [28,29] and—If effective in murine models.

In summary, our data give novel immunologic and mechanistic insights into processes in DCs and PBMCs infected with non- or complement-opsonized HIV-1 by sole treatment with the P80 natural essence. Besides exerting an efficient antiviral effect in DCs, the natural compound illustrates an adjuvant effect in terms of improving DC maturation and co-stimulatory capacity upon exposure to HIV or HIV-C and also mediates a sustained p38 MAPK phosphorylation. These findings might be exploited for future therapeutic options to improve antiviral or adjuvant immune responses via not yet considered host mechanisms in DCs against non- as well as complement-opsonized HIV-1.

## Supplementary Materials

The following are available online at https://www.mdpi.com/article/10.3390/vaccines9090976/s1, Figure S1. P80 natural essence blocks DC infection of non-opsonized HIV-1 independent of the mode of addition. DCs were loaded with either HIV that was opsonized with 1% P80 prior addition (HIV_P80 pre-opsonized, cells were pre-incubated with 1% P80 prior HIV addition (1% P80 pre_HIV) or infected with HIV prior addition of 1% P80 (HIV_1% P80 post). Compared to high DC infection using HIV, all treatments highly significantly reduced productive infection in DCs. Statistical differences were analysed using GraphPad Prism software and one-way ANOVA with Dunnett’s post-test. **** p < 0.0001. Figure S2. P80 natural essence enhances maturation in HIV-exposed DCs. Not only surface expression of the co-stimulatory marker CD86, but also the characteristic DC maturation marker was elevated in DCs pre-incubated with P80 prior to addition of HIV-1 (grey) compared to HIV-exposed DC (green). In blue, immature control DC are depicted and in yellow, DC is exposed to P80 only. Data were analysed using Flow Logic software and experiments were independently repeated at least 3 times.
