# Supplementary material for: P80 Natural Essence Exerts Efficient Anti-HIV-1- as Well as Adjuvant Effects in DCs
Source: Vaccines (Basel). 2021 Aug 31;9(9):976. doi: 10.3390/vaccines9090976 (PMC8472994; doi:10.3390/vaccines9090976)
Supplement: Supplementary file 1 [file vaccines-09-00976-s001.zip › vaccines-1193272-supplementary.pdf]

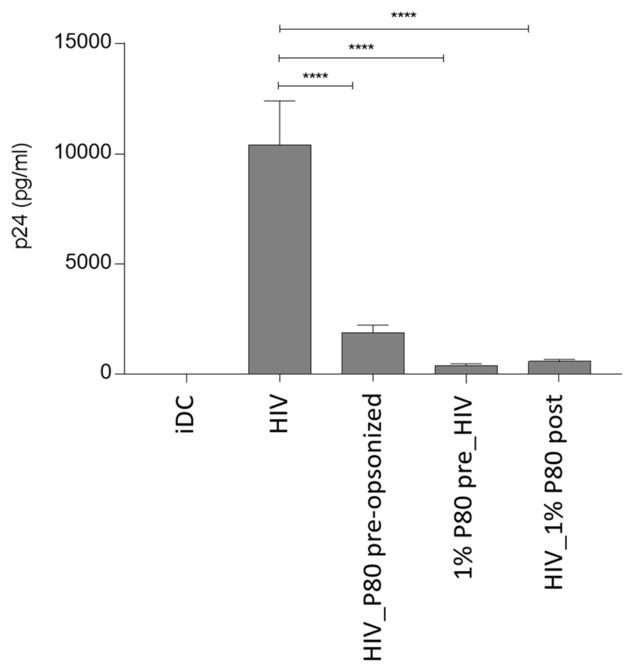

Figure S1. P80 natural essence blocks DC infection of non-opsonized HIV-1 independent on the mode of addition. DCs were loaded with either HIV that was opsonized with 1% P80 prior addition (HIV_P80 preopsonized, cells were pre-incubated with 1% P80 prior HIV addition (1% P80 pre_HIV) or infected with HIV prior addition of 1% P80 (HIV_1% P80 post). Compared to high DC infection using HIV, all treatments highly significantly reduced productive infection in DCs. Statistical differences were analysed using GraphPad Prism software and one-way ANOVA with Dunnett´s posttest. **** $p < 0.0001$.

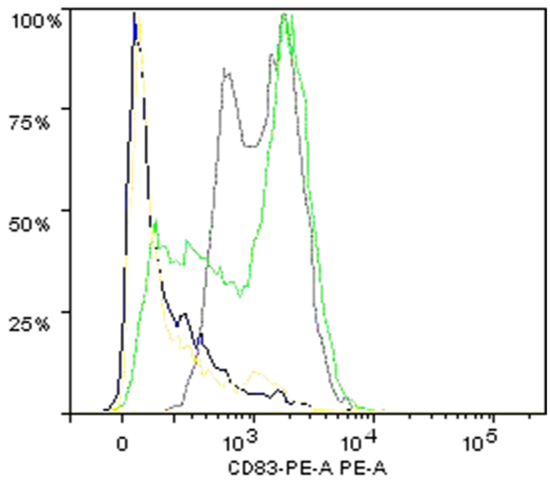

Figure S2. P80 natural essence enhances maturation in HIV-exposed DCs. Not only surface expression of the co-stimulatory marker CD86, but also the characteristic DC maturation marker was elevated in DCs pre-incubated with P80 prior addition of HIV-1 (grey) compared to HIV-exposed DC (green). In blue, immature control DC are depicted and in yellow, DC exposed to P80 only.

Data were analysed using Flow Logic software and experiments were independently repeated at least 3 times.
